# SMARTPHONES CAPTURE DYNAMICS OF DEMENTIA SPOUSAL CAREGIVER INTERACTIONS: IMPLICATIONS FOR MHEALTH

**DOI:** 10.1093/geroni/igae098.1411

**Published:** 2024-12-31

**Authors:** Christopher Fagundes

**Affiliations:** Rice University, Houston, Texas, United States

## Abstract

Dementia spousal caregivers experience higher levels of loneliness than age-match comparisons. Lonelier dementia spousal caregivers report more depression and less life satisfaction; people with dementia who have lonelier spousal caregivers report lower life satisfaction and more depression than those cared for by less lonely spousal caregivers; however, caregivers are unaffected by the degree of loneliness experienced by their spouse with dementia. Interventions designed to address loneliness in caregivers could improve life satisfaction for both dyad members. Unfortunately, little is known about the patterns and temporal dynamics of loneliness in caregivers and how context, interpersonal, and intrapersonal processes buffer or exacerbate experiences of loneliness. This lack of knowledge hampers our ability to intervene; for example, despite the enthusiasm for mHealth technology to deliver just-in-time, adaptive interventions, our gaps in understanding loneliness dynamics thwart empirically informed intervention development. In this presentation, we will present data to fill this gap in the literature using smartphones to capture loneliness dynamics in caregivers and elucidate how these dynamics impact dyads across a 14-day ecological momentary assessment protocol. We will present data on how caregivers’ sense of relational security impacts loneliness and life satisfaction after dyadic interactions. For example, caregivers’ sense of relational security determined whether performing caregiving tasks involving dyadic interactions increased or decreased subsequent loneliness (b=.01, SE=.02, p<.05). We conclude by discussing ways in which smartphones can be used to capture momentary patterns of mood and behavior, and how this data could inform mHealth interventions to improve the lives of both dyad members.

